# *In Silico* Identification and Characterization of *N-Terminal Acetyltransferase* Genes of Poplar (*Populus trichocarpa*)

**DOI:** 10.3390/ijms15021852

**Published:** 2014-01-27

**Authors:** Hang-Yong Zhu, Chun-Ming Li, Li-Feng Wang, Hui Bai, Yan-Ping Li, Wen-Xi Yu, De-An Xia, Chang-Cai Liu

**Affiliations:** 1 State Key Laboratory of Tree Genetics and Breeding, Northeast Forestry University, 26 Hexing Road, Harbin 150040, China; E-Mail: zhhy504@hotmail.com; 2 Forestry Research Institution of Heilongjiang Province, Harbin 150081, China; E-Mails: lichunming_lcm@163.com (C.-M.L.); baihui1979@163.com (H.B.); lkykjc@126.com (W.-X.Y.); 3 Faculty of life Science and Technology, Mudanjiang Normal University, 191 Wenhua Street, Mudanjiang 157012, China; E-Mails: swxwlf@126.com (L.-F.W.); swxlyp@126.com (Y.-P.L.); 4 Laboratory for Chemical Defense and Microscale Analysis, Hubei Nanxing General Chemical Factory, Zhijiang 443200, China

**Keywords:** acetyltransferase, *N*α-acetylation, genome identification, woody plants, phylogenetic analysis

## Abstract

*N*-terminal acetyltransferase (Nats) complex is responsible for protein *N*-terminal acetylation (*N*α-acetylation), which is one of the most common covalent modifications of eukaryotic proteins. Although genome-wide investigation and characterization of Nat catalytic subunits (CS) and auxiliary subunits (AS) have been conducted in yeast and humans they remain unexplored in plants. Here we report on the identification of eleven genes encoding eleven putative Nat CS polypeptides, and five genes encoding five putative Nat AS polypeptides in *Populus*. We document that the expansion of *Nat CS* genes occurs as duplicated blocks distributed across 10 of the 19 poplar chromosomes, likely only as a result of segmental duplication events. Based on phylogenetic analysis, poplar Nat CS were assigned to six subgroups, which corresponded well to the Nat CS types (CS of Nat A–F), being consistent with previous reports in humans and yeast. *In silico* analysis of microarray data showed that in the process of normal development of the poplar, their *Nat CS* and *AS* genes are commonly expressed at one relatively low level but share distinct tissue-specific expression patterns. This exhaustive survey of *Nat* genes in poplar provides important information to assist future studies on their functional role in poplar.

## 1. Introduction

Protein *N*-terminal acetylation (*N*α-acetylation) is one of the most common covalent modifications of eukaryotic proteins, in which an acetyl group is transferred from acetyl-CoA to the α-amino group of protein *N*-terminal residues [1–4]. *N*α-acetylation of proteins might act as a destabilization signal for some yeast proteins or stabilizer mediated degradation by blocking *N*-terminal ubiquitination [5,6]. Unlike most other protein modifications, *N*α-acetylation is irreversible [7,8]; it mainly occurs cotranslationally on nascent polypeptide chains and almost all *N*α-acetylation is catalyzed by the action of ribosome associated *N*-terminal acetyltransferase (Nats) complex in eukaryotes [8].

Currently, six types of Nats (NatA–F) complexes conserved from yeast to humans are responsible for these Nα-acetylation events: each of the three major Nats, NatA, NatB and NatC contain a catalytic subunit, and one or two auxiliary subunits, whereas NatD, NatE and NatF are composed of only one catalytic subunit [8,9]. Each type of Nats appears to acetylate a distinct subset of substrates [8,10], and there are also crossing subsets of substrates between particular Nats [9]. Evidence has indicated that Nats are involved in a number of cellular processes in the lower eukaryotes, while NatA, NatB and NatC are associated with cell cycle arrest or apoptosis, NatE with sister chromatid cohesion, and NatF with normal chromosome segregation in higher eukaryotes [9]. Although these considerable advances have been made in exploring components and in the function of Nats in yeast and humans, such in-depth study has not been directed towards plants, especially for woody plants.

The entire gene encoding catalytic or auxiliary subunits of NatA–NatF have been identified and described in yeast and humans (Table 1) [9,10]. However, there is still no systematic and comprehensive characterization of Nats in poplar. In order to explore all genes encoding Nat catalytic subunits (CS) and auxiliary subunits (AS) in poplar, the complete *Populus trichocarpa* genome was investigated using the method of domain search. Here, we exhibit the identification and analysis of Nats and their respective genes in *Populus trichocarpa*. As we know, this is the first systematic characterization of all genes encoding CS and AS of Nat in a single woody plant genome, and represents the basis for future studies on the composition and function *in vivo* of each poplar Nat.

## 2. Results and Discussion

### 2.1. Identification and Characterization of Genes Encoding Nat Subunits in *Populus*

Before this work six types of Nats (NatA–F) had been found and identified in a few eukaryotes, amongst which NatA, NatB and NatC complex were composed of AS and CS, whereas NatD, NatE and NatF complex were only composed of CS [9]. However, it still remained unexplored whether there were corresponding genes encoding similar AS and CS orthologs of Nats in the genome of the single woody plants. In order to precisely obtain all members of each type of Nat complex orthologs in *Populus*, domain files representing subunits of individual types [11] were exploited as queries to identify the AS and CS orthologs of Nat complex in the *P. trichocarpa* genome [12]. As a result, a total of 11 non-redundant putative *Nat CS* genes were identified as significantly encoding the CS domain of individual Nats, amongst which except for the CS of NatD encoded by one gene, the CS of the remaining Nats (NatA, B, C, E and F) were respectively encoded by two paralogous genes (Table 1). There are five non-redundant putative *AS* genes identified as significantly encoding the AS domain of individual Nats, with one encoding the AS of NatB, one encoding the AS I of NatC, one encoding the AS II of NatC, and two encoding the AS of NatA (Table 1). They were designated as novel simplified nomenclature according to a previous study [13], for example, the two Nat CS of *P. trichocarpa* were respectively named as Ptr Naa10p and Ptr Naa11p (Table 1). Since such information had not been characterized in other model plants, an extended domain search across the Arabidopsis protein sequence database (http://www.arabidopsis.org/), was performed to identify the AS and CS of *Arabidopsis* Nats. It was found that, although the *Arabidopsis* genome also contains the entire genes encoding CS or AS of Nat complex (NatA–F), few paralogous genes were found to encode the same one CS of Nats, which is consistent with the occurrence in yeast and humans [14,15].

In other words, we found that both Arabidopsis and poplar genomes contain the full Nat system composed of NatA–F. Most of the Nat catalytic subunits in poplar exist as two paralogous isoforms: Ptr Naa10p and Ptr Naa11p for the poplar NatA CS, Ptr Naa20p and Ptr Naa21p for NatB CS, Ptr Naa30p and Ptr Naa31p for NatC CS, Ptr Naa50p and Ptr Naa51p for NatE CS, as well as Ptr Naa60p and Ptr Naa61p for NatF CS (Table 1), while only NatD CS exists as a single protein, Ptr Naa40p (Table 1). In comparison with other eukaryotes, no Nat CS contains paralogous isoforms in yeast, only one NatA CS contains paralogous isoforms (*i.e*., Naa10p and Naa11p) in humans and one NatF CS contains paralogous isoforms (Ath Naa60p and Ath Naa61p) in *Arabidopsis* [14]. These results above implied that the genes encoding Nat CS in poplar have expanded. This expansion, often present in a large number of *Populus* multi-gene families, could have occurred from multiple gene duplication events, involving in segmental duplication and tandem duplication events [12]. However, it was very necessary for our further understanding of their function to identify in the expansion which events play a critical role. It has been suggested that the presence of more *Nat CS* genes in the *Populus* genome might reflect a greater requirement for acetylation of proteins. In summary, our *in silico* identification showed that the *P. trichocarpa* genome not only contains the entire genes encoding CS or AS of Nat complex (NatA–F), but also the expansion of the genes encoding Nat CS is different from those of other known eukaryotes.

### 2.2. Chromosomal Location and Duplication of *Nat CS* Gene in *Populus*

**Figure 1.**
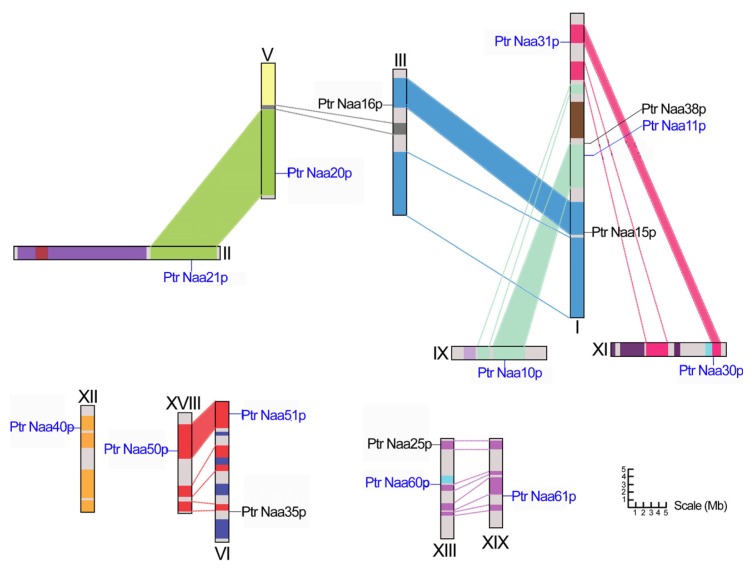
Chromosomal location of the *Populus N*-terminal acetyltransferase (Nat) catalytic subunit (*CS*) and auxiliary subunit (*AS*) genes. All sixteen genes are mapped to the 11 of nineteen Linkage Groups (LG). The schematic representation of genome-wide chromosome organization arising from the whole-genome duplication event in *Populus* was obtained from the study of Tuskan and its co-workers [12]. Segmental duplicated homologous regions are shown with the same color. Only the duplication blocks containing *Nat CS* and *AS* genes are connected with lines in shaded colors. The scale at the bottom represents a 5 Mb chromosomal distance.

To explore the reasons for the expansion of *Nat CS* genes in the *Populus* genome, wide-genome chromosomal location was performed in this study. *In silico* mapping of the gene loci showed that, these genes encoding CS and AS of Nats in *P. trichocarpa*, were distributed across 11 of 19 Linkage Groups (LGs) (Table 1 and Figure 1). Eleven *Nat CS* genes were distributed across 10 of the 19 LGs, while five *Nat AS* genes across four of the 19 LGs. The distribution of the *Nat CS* genes among 10 LGs appears to be relatively even: LG II, V, VI, IX, XI, XII, XIII, XVIII and XIX individual have only one *Nat CS* gene, while LG I contains two *Nat CS* genes (Ptr Naa11p and Ptr Naa31p) in which high density cluster within a 20 kb fragment has not been formed. The distribution of *Nat AS* genes among four LGs also seems to be relatively even: LG III, VI, and XIII respectively have one *AS* gene, two genes (Ptr Naa15p and Ptr Naa38p) that are far apart were located in the same LG I (Figure 1). The results above showed the absence of tandem duplication events present in the process of expansion of poplar *Nat CS* genes.

Previous analysis of the *Populus* genome has identified the presence of paralogous segments caused by the whole-genome duplication event in the Salicaceae (salicoid duplication), which occurred 65 million years ago and significantly contributed to the amplification of many multi-gene families [12]. To determine the possible relationship between the *Nat CS* genes and their paralogous segments, the *Populus Nat CS* genes were mapped to the duplicated blocks of *P. trichocarpa* established in the studies of Tuskan and his coworkers [12]. The distribution of *Nat CS* genes relative to the duplicated blocks is illustrated as in Figure 1. It was found that nine of all the eleven mapped *Nat CS* genes (82%) are located in duplicated blocks. Four duplicated pairs (PtrNaa10/11p, PtrNaa20/21p, PtrNaa30/31p and PtrNaa50/51p) are each located in a pair of paralogous blocks created by the whole-genome duplication event, and can be considered as a direct result of the segmental duplication event (Figure 1). One duplicated pair (PtrNaa40) harbored *Nat CS* genes on only one of the blocks and lacks corresponding duplicates, suggesting that the loss event of its corresponding paralogous genes would have occurred after the segmental duplication events (Figure 1). The findings support the result that the most abundant gene losses in eukaryotes occur following the whole genome duplication [16]. In addition, one pair of PtrNaa60p and PtrNaa61p that are the NatF orthologs corresponding to new identified human Naa60p [9], are respectively located in non-duplicated blocks of LG XIII and XIX. However, between the two chromosomes, there are numerous homologous genome blocks, suggesting that the expansion of the poplar *NatF CS* gene could have resulted from other duplicated events.

The segmental duplication as well as the tandem duplication events were thought to be the main factors in contributing to the expansion of the gene family in *Populus* [12]. However, in our study no tandem duplication events were found, indicating that the presence of the segmental duplication events might be single events contributing to the expansion of the *Populus Nat CS* gene family. In a different way, the two events in *Populus* genome had also been shown to contribute to the expansion of NAC [17] and GLUC [12] *etc*. gene families. Here, the *Populus Nat CS* gene family has been preferentially retained at a rate of 82%, while in the *Populus* genome, only about one-third of putative genes are retained in duplicated blocks resulting from the whole genome duplication events [12]. The high retention rate of duplicated genes has also previously been documented in other *Populus* gene families [17–20].

### 2.3. Phylogenetic Analysis of *Nat CS*

**Figure 2.**
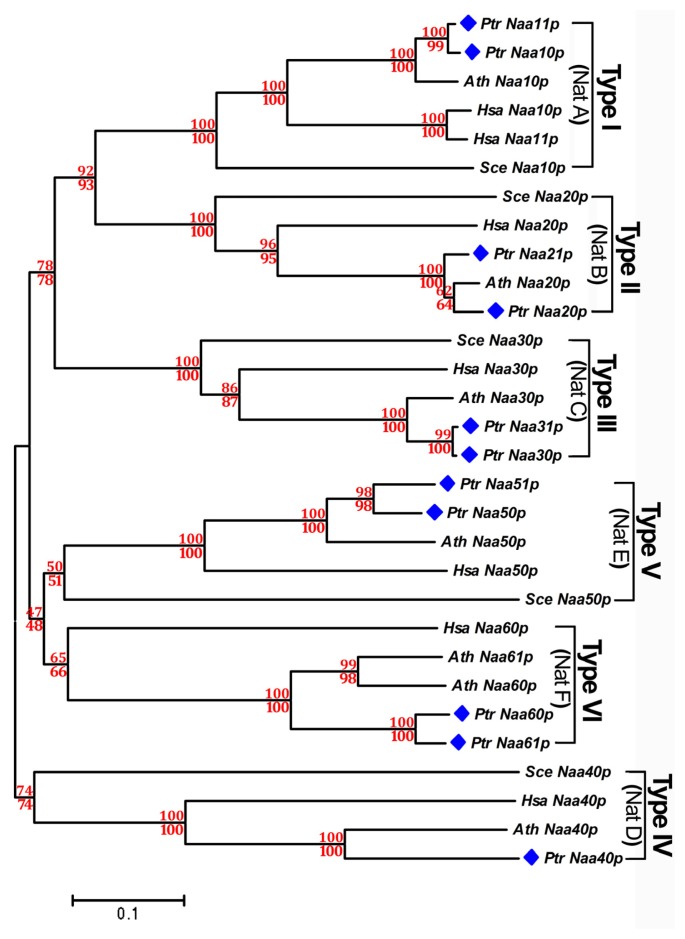
Phylogenetic relationships of poplar Nat CS proteins. Neighbor-Joining bootstrap and Minimum Evolution values for clans supported above the 47% level were respectively indicated above and below the branches in red font. The blue diamonds are highlighted in the front of all Nat CS subtypes from *Populus*. All poplar Nat CS and AS protein names and their individual corresponding ID number for phylogenetic analysis are listed as in Table 1. Sce Naa10p (P07347); Sce Naa20p (Q06504); Sce Naa30p (Q03503); Sce Naa40p (Q04751); Sce Naa50p (Q08689); Hsa Naa10p (P41227); Hsa Naa11p (Q9BSU3); Hsa Naa20p (P61599); Hsa Naa30p (Q147X3); Hsa Naa40p (Q86UY6); Hsa Naa50p (Q9GZZ1); Hsa Naa60p (Q9H7X0); Ath Naa10p (AT5G13780); Ath Naa20p (AT1G03150); Ath Naa30p (AT2G38130); Ath Naa40p (AT1G18335); Ath Naa50p (AT5G11340); Ath Naa60p (AT5G16800); Ath Naa61p (AT3G02980).

**Figure 3.**
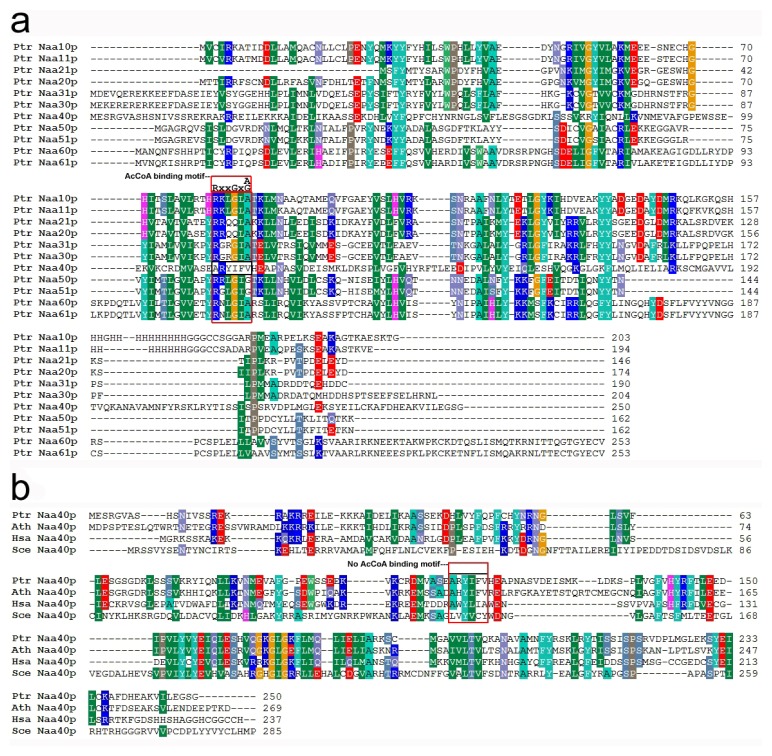
Amino acid sequence alignment. (**a**) Amino acid sequence alignment of all predicted Nat CS from poplar; (**b**) Amino acid sequence alignment of poplar NatD catalytic (Ptr Naa40p) subunit with its counterparts from *Arabidopsis*, yeast and humans. Gaps are introduced to ensure maximum identity. Color shading represents 70% identical residues among the sequences. The consensus acetyl coenzyme A (AcCoA) binding motif sequence RxxGxG/A, where x can be any amino acid, is boxed (red). The identifiers of the Nat CS proteins from poplar are shown in Table 1. Ath Naa40p (AT1G18335); Hsa Naa40p (Q86UY6); Sce Naa40p (Q04751).

To gain insight into the evolutionary relationship of the *Nat CS* genes family, an unrooted tree was respectively generated by both Minimum-Evolution methods using MEGA 5.0 [21] and Neighbor-Joining [22] based on complete protein sequences of all type of *Nat CS* genes in *Populus*, *Arabidopsis*, human and yeast. The tree topologies generated by the two methods were comparable without modifications at branches, and were supported by their high bootstrap values of >47, suggesting that we had constructed a reliable unrooted tree topology, in which the 30 *Nat CS* were grouped into six distinct clans including Type I, Type II, Type III, Type IV, Type V and Type VI (Figure 2). The six distinct types generated by their evolutional divergence corresponded well to the Nat CS subgroups (CS of Nat A–F) (Figure 2), which is consistent with previous reports in humans and yeast [9]. Both Minimum-Evolution and Neighbor-Joining analyses suggest an association of the Type I, II, III, V and VI Nat CS proteins to the exclusion of the Type IV Nat CS proteins (Figure 2). It could be explained well by previous evidence that the apparent amino acid sequence difference between NatD CS and other types of Nat CS from yeast and humans had occurred in the acetyl coenzyme A (AcCoA) binding motif “RxxGxG/A”, which is a sequence feature of the *N*-acyltransferase family [23]. To expand this evidence, amino acid sequence alignment among all types of poplar Nat CS (Figure 3a), as well as between poplar NatD CS with NatD counterparts from yeast, humans and *Arabidopsis* was performed (Figure 3b). It was found that the AcCoA binding motif RxxGxG/A is present in the CS of each poplar NatA, NatB, NatC, NatE and NatF except for poplar NatD CS (Naa40p) (Figure 3a), whereas the absence of this motif occurred in all CS of NatD (Naa40p) from *Arabidopsis*, poplar, yeast and humans (Figure 3b).

The analyses group Type I, III, V and VI isoforms of *Populus* (Ptr Naa10/11p, PtrNaa30/31p, PtrNaa50/51p and PtrNaa60/61p), Type I isoforms of human (Hsa Naa10/11p) and Type VI isoforms (Ath Naa60/61p) were assigned within their respective clades. In addition, the groupings of Type II isoforms of *P. trichocarpa* (PtrNaa20p and PtrNaa21p) suggest additional recent duplication events within these lineages. This evidence further supports the expansion of the *Nat CS* gene family in the *Populus* genome caused by segmental duplication events.

### 2.4. Tissue Location of *Nat CS* and *AS* Gene Expression in *Populus*

**Figure 4.**
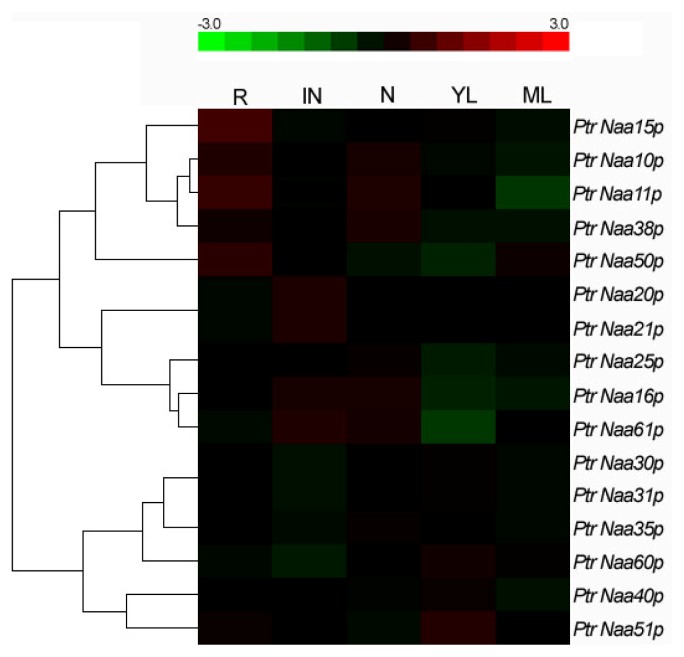
Relative transcript abundance profiles of *Populus Nat CS* and *AS* genes across different tissues. A heat map displaying the transcript abundance is produced here using the genome-wide microarray data generated by Wilkins and coworkers [26]. The transcript abundance levels for the *Populus Nat CS* and *AS* genes were clustered using hierarchical clustering based on the Pearson correlation. The color scale at the bottom of each dendrogram represents log2 expression values, green color represents low level, red color represents high level of transcript abundances and black color represents no transcript expression. The symbols represent as follows: R, root; IN, internodes; N, nodes; YL, young leaf; ML, mature leaf.

Although numerous studies prior to this work were mainly focused on the expression, composition and function of Nats from several eukaryotes, such as yeast, mouse and human [24], such a systematic investigation had not yet been conducted in plants, especially for woody plants. Publicly available microarray data has often been considered as a reliable means of studying gene expression profiles [25]. To investigate the expression pattern of all poplar *Nat CS* and *AS* genes, the poplar Affymetrix microarray data [26] were reorganized in the *Populus* Genome Integrative Explorer (PopGenIE) [27]. All 16 poplar *Nat* genes including 11 *CS* and five *AS* genes have their corresponding transcript ID in the dataset and their expression profiles are displayed as shown Figure 4. It was found that expression of poplar *Nat AS* and *CS* genes in all five tissues were commonly low level in the process of normal development, but they also showed distinct tissue-specific expression patterns that were preferentially expressed in root (R), internode (IN), node (N) and young leaf (YL) while few in mature leaf (ML) (Figure 4). The highest expression level was found in the R, IN and YL, suggesting that in these tissues *N*-terminus of more proteins might be needed to undergo *N*α-acetylation catalyzed by Nats for certain signal transmissions. The three genes encodingPtr Naa10p, Ptr Naa11p and Ptr Naa15p combined into Ptr NatA complex [28], have significantly similar expression patterns and high-level expression is mostly present in R and N (Figure 4). The expression profile of Ptr Naa20p, Ptr Naa21p and Ptr Naa25p genes encoding Ptr NatB complex showed also relatively consistently that transcript accumulation is focused on IN, few transcript expressions are focused on R, N, YL and ML. Furthermore, it was notable that consistent expression patterns were also found in the three genes encoding Ptr Naa30p, Ptr Naa31p and Ptr Naa35p combined into Ptr NatC complex, which have almost no expression in all five tissues. The evidence that poplar *Nat CS* and *AS* genes combined into the same Nat complex share similar expression patterns across tissues, seems likely to contribute to fast assembly from their individual subunit combination into active Nat complex.

## 3. Experimental Section

### 3.1. Acquisition or Establishment of Hidden Markov Model (HMM) Profile Files

Hidden Markov Model (HMM) profile files of Mdm20 (PF09797) and Mak10 (PF04112) subunits were known and loaded from the Pfam database (http://pfam.sanger.ac.uk/). HMM profile files representing the other nine Nat subunits were unexplored and needed to be established. Firstly, these known protein sequences representing each subunit from various organisms were respectively extracted from the UniProt database (http://www.uniprot.org), and then were aligned using the ClustalW program to produce Stockholm files [29]. Subsequently, their HMM profile files were respectively in-house established using the hmmbuild command of the HMMER (v 3.0) software [11].

### 3.2. Domain Profile Search

The genes encoding each Nat subunit of *Populus* and *Arabidopsis* were *in silico* identified by the method of Domain profile search. HMM profile files representing each Nat ortholog subunit were searched against the poplar protein database [12] using the hmmer search command of the HMMER (v 3.0) software with the sequence reporting threshold parameter (*E*-value ≤ 1000) [11]. In the same manner, these above HMM profile files were searched against the *Arabidopsis* protein database [14].

### 3.3. Chromosomal Location and Phylogenetic Analysis

The genes encoding Nat subunits (CS and AS) were located in the genome of *Populus trichocarpa* using NCBI map viewer (http://www.ncbi.nlm.nih.gov/projects/mapview/). Identification of duplicated regions between chromosomes was completed as described in Tuskan *et al*. [12]. The tandem gene duplication in poplar was determined according to the criteria that five or fewer gene loci occurred within a range of 100 kb distance [17,18,30–32].

The total 30 Nat CS protein sequences of *Populus*, *Arabidopsis*, human and yeast were obtained from the Nr protein database of NCBI (http://www.ncbi.nlm.nih.gov/) by batch extraction. Alignments of the full-length protein sequences were performed using the ClustalW program in BioEdit software with default parameters [33]. Based on these aligned sequences, the unrooted phylogenetic trees were constructed using MEGA 5.0 software [21,34], by both Neighbor-Joining method [22] and Minimum Evolution method with parameters (*p*-distance and partial deletion). The reliability of the phylogenetic tree was estimated using a bootstrap value with 1000 replicates.

### 3.4. *In silico* Microarray Analysis

Transcript IDs corresponding to the individual poplar *Nat* gene were retrieved from Popgenie 2.0 (http://popgenie.org/), in which a set of integrated online tools could be applied to facilitate the exploration of genes and gene function in *Populus*. The transcript relative abundance values of all poplar Nat genes from various tissues were obtained from the poplar transcript abundances datasets [26], whose data originated from the NCBI Gene Expression Omnibus (accession number: GSE13990). A set of integrated online tools including gene search, experiment search and ePlant expression viewer were successively applied to extract *Nat* gene expression values in special tissues. Dendrogram and heat map for display expression pattern were obtained using Cluster 3.0 [35] for normalizing and hierarchical clustering with average linkage based on Pearson coefficients, followed by Java Tree-View 1.1 program [36] for visualizing the analyzing datasets.

## 4. Conclusions

Considerable research efforts have been conducted into the characterization of Nat complexes in yeast and humans, but such effort has not yet been directed towards plants, especially for woody trees. In this work, the above issues were addressed using the method of genome-wide identification and *in silico* analysis. Unlike most of eukaryotes, the expansion of encoding *Nat CS* genes was found in the poplar genome which could have resulted from segmental duplication events. Although the poplar has more Nats than yeast and humans do, it also contains the entire genes encoding CS or AS of Nat complex (NatA–F), suggesting that the *N*α-acetylation patterns and the Nat machinery should be similar between the poplar and other higher eukaryotes. This comprehensive analysis is an important starting point for future efforts to elucidate the functional role of all Nat complex proteins in poplar.

## Figures and Tables

**Table 1.** All identified *N*-terminal acetyltransferase (Nat) genes (*CS* and *AS*) and putative encoded poplypeptides present in *Populus trichocarpa* genome.

Type	JGI gene and protein ID	Transcript ID		Chromosome location	Protein products	
	
		NCBI REFseq	*Populus* genome V2.2		Protein ID	Novel simplified nomenclature
*NatA CS* a	650021	XM_002314022.1	POPTR_0009s06150	LG_IX:6944007–6945077(−)	XP_002314058.1	Ptr Naa10p
*NatA CS*	641307	XM_002298379.1	POPTR_0001s26920	LG_I:18982354–18983685 (+)	XP_002298415.1	Ptr Naa11p
*NatA AS* b	548659	XM_002299594.1	POPTR_0001s17830	LG_I:9952442–9966294 (−)	XP_002299630.1	Ptr Naa15p
*NatA AS*	553694	XM_002304144.1	POPTR_0003s05540	LG_III:4692360–4705382 (−)	XP_002304180.1	Ptr Naa16p
*NatB CS*	818659	XM_002307550.1	POPTR_0005s23200	LG_V:14531524–14534737 (−)	XP_002307586.1	Ptr Naa20p
*NatB CS*	643297	XM_002300805.1	POPTR_0002s05290	LG_II:3418242–3421271 (+)	XP_002300841.1	Ptr Naa21p
*NatB AS*	571859	XM_002319920.1	POPTR_0013s14900	LG_XIII:12260671–12271953 (−)	XP_002319956.1	Ptr Naa25p
*NatC CS*	727122	XM_002316966.1	POPTR_0011s14270	LG_XI:13438711–13441426 (+)	XP_002317002.1	Ptr Naa30p
*NatC CS*	642436	XM_002298895.1	POPTR_0049s00200	LG_I:32126776–32129356 (+)	XP_002298931.1	Ptr Naa31p
*NatC AS I*	560565	XM_002308020.1	POPTR_0006s06370	LG_VI:3978171–3986294 (+)	XP_002308056.1	Ptr Naa35p
*NatC AS II*	641478	XM_002299954.1	POPTR_0001s28460	LG_I:20275848–20278373 (−)	XP_002299990.1	Ptr Naa38p
*NatD CS*	729076	XM_002318277.1	POPTR_0012s03830	LG_XII:300904–304114 (−)	XP_002318313.1	Ptr Naa40p
*NatE CS*	737117	XM_002324238.1	POPTR_0018s01280	LG_XVIII:5217292–5220254 (+)	XP_002324274.1	Ptr Naa50p
*NatE CS*	654093	XM_002308604.1	POPTR_0006s26500	LG_VI:16869902–16872551 (+)	XP_002308640.1	Ptr Naa51p
*NatF CS*	834607	XM_002319219.1	POPTR_0013s07770	LG_XIII:7060330–7064827 (+)	XP_002319255.1	Ptr Naa60p
*NatF CS*	665408	XM_002325352.1	POPTR_0019s07740	LG_XIX:4276553–4280210 (+)	XP_002325388.1	Ptr Naa61p

a CS denotes catalytic subunit of Nat;

b AS represents auxiliary subunit of Nat.
